# Outcome of Topical Steroid Application in Children with Non-retractile Prepuce

**DOI:** 10.34763/devperiodmed.20182201.7174

**Published:** 2018-04-12

**Authors:** Deepa Makhija, Hemanshi Shah, Charu Tiwari, Pankaj Dwiwedi, Suraj Gandhi

**Affiliations:** 1 Departament of Pediatric Surgery, T.N.M.C, Mumbai, India

**Keywords:** phimosis, physiological, pathological, steroid, topical

## Abstract

True phimosis is overdiagnosed due to the failure to distinguish it from physiological phimosis, which is a normal developmental non retractability of the foreskin. The non-retractile prepuce in children is a cause of parental anxiety and concern. This leads to the majority of the children undergoing surgical procedures. Pathological phimosis needs to be differentiated from physiologic phimosis to avoid unnecessary circumcision. In recent years, topical steroid application use in cases of non-retractile prepuce has shown a good success rate and is well accepted by the parents. It has low risks, is cost effective and avoids anaesthetic and surgical complications.

This is an observational study of 100 children with non-retractile foreskin who were managed by local application of topical steroid cream (0.1% Mometasone) over a period of 6 weeks. The non-retractibility was classified according to Kikiro’s classification. These patients were analyzed on the basis of age at presentation, complaints at the first presentation, grade of phimosis at first presentation (as per Kikiro’s classification), results of the topical steroid application as assessed at 6 weeks after starting application and after stopping of the steroid administered for 6 weeks. The results were analyzed on the basis of the resolution of symptoms and the decrease in Kikiro’s grade. Those patients in whom there was no response to treatment or who developed recurrence after stopping steroid treatment underwent circumcision. A total of 19 patients required surgical intervention in the form of circumcision. The use of topical steroids yields satisfactory results in patients with a non-retractile prepuce. It could be a first-line treatment for management in such cases and is an effective alternative designed to avoid unnecessary circumcision.

## Introduction

Phimosis is the non-retraction of the prepuce. It is physiological in younger children due to adhesions between the prepuce and glans penis [1]. Most patients referred due to phimosis are actually suffering from the physiological type of non-retractability. Physiological phimosis is widely prevalent in male newborns. However, the degree of preputial retractability increases with age and the stage of preputial separation varies greatly among individuals^2^. It is termed pathologic when associated with local or urinary complaints attributed to the scarred prepuce. The difficulty in differentiating between physiological and pathological phimosis leads to undue concern and anxiety among parents and unnecessary referrals.

In the past decades, the first line of treatment of non-retractile prepuce was circumcision. This operative intervention is not without adverse effects and has a large economic impact. The only reasonable indication for circumcision, i.e. a pathological phimosis, affects about 0.6% of boys, with a peak in incidence at the age of 11 years, and is rarely encountered before the age of 5 years [2]. With the advent of newer effective and safe medical and conservative techniques, circumcision is gradually getting outmoded.

In the 90’s, topical steroids were introduced as a nonsurgical alternative for the treatment of phimosis^3^. Their potential advantages are: less trauma, lower cost, avoidance of anaesthesia and surgical complications like hemorrhage, pain and infection [3]. The success rates of steroids in phimosis as reported in the literature ranges from 65% to almost 90% [3]. The aim of topical corticosteroid treatment is to reduce skin tightening around the tip of the penis. This offers a relatively less invasive treatment and may limit the need for surgery among the majority of boys [4].

## Materials and methods

**Fig. 1:**
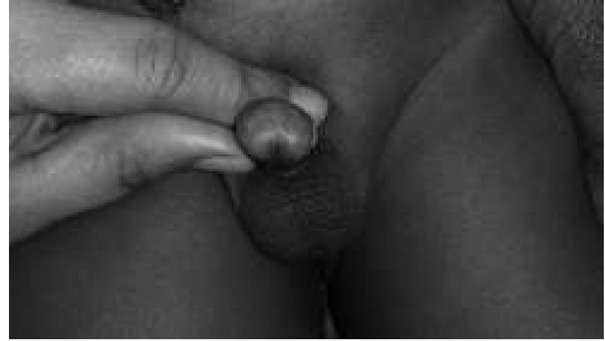
Kikiros Grade 0.

This is a retrospective observational study. With informed consent, we retrospectively analyzed the data of consecutive 100 paediatric patients (age less than 12 of balanoposthesis/a history of recurrent urinary tract years) who presented with a non-retractile prepuce at our infections were excluded from the study. At the first visit, Paediatric Surgery Out Patient Department, a tertiary a clinical examination was performed and phimosis was care centre, from August 2013 to February 2017. Patients evaluated according to the classification of Kikiros and who had previously taken treatment or who had signs Woodward (Table I) [5]; (Figure 1 to Figure 6).

**Fig. 2:**
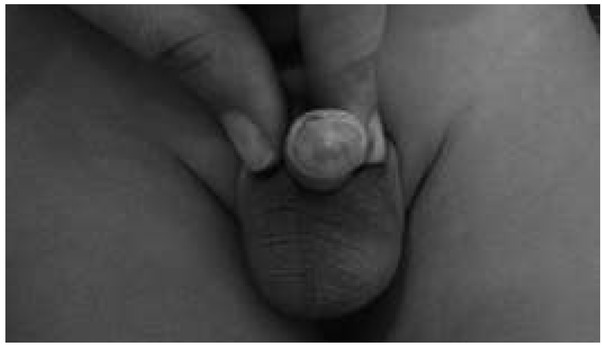
Kikiros Grade 1.

**Fig. 3:**
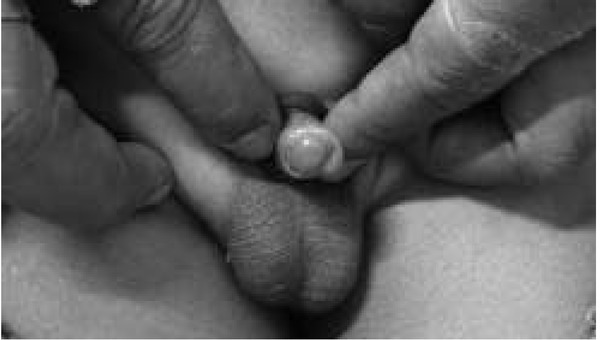
Kikiros Grade 2.

**Fig. 4:**
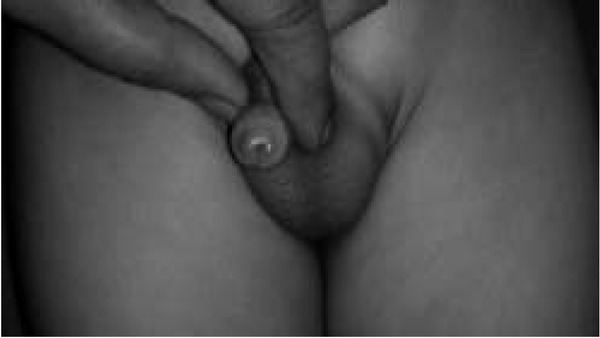
Kikiros Grade 3.

**Fig. 5:**
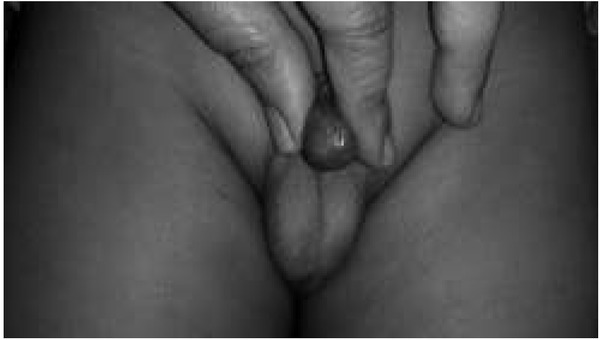
Kikiros Grade 4.

**Fig. 6:**
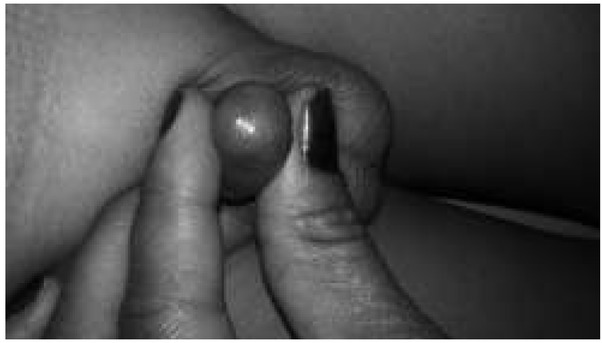
Kikiros Grade 5.

**Table I:** Grades Of Retractability Of The Foreskin According To Kikiros et al.

Grade	Description
0	Full retraction, not tight behind glans, or easy retraction limited only by congenital adhesions to the glans
1	Full retraction of foreskin, tight behind the glans
2	Partial exposure of glans, prepuce (no congenital adhesions) limiting factor
3	Partial retraction, meatus just visible
4	Slight retraction, but some distance between tip and glans, i.e., neither meatus nor glans can be exposed
5	Absolutely no retraction

### Technique for application of the topical steroid ointment

The use of the topical steroid ointment was explained and taught to parents before the initiation of the treatment at home. The parents were informed about the possible local side effects of the steroid ointment, such as striae, pigmentation changes, telangiectasia, and hypertrichosis. The technique involved a warm sitz bath for 20 minutes followed by the application of topical mometasone cream after the gentle retraction of the prepuce. No attempt was made to forcibly retract the prepuce, so as to avoid splitting and bleeding of the foreskin. The application was done three times a day for a period of six weeks. No occlusive dressing was used. Patients were followed up every 15 days.

### Evaluation of the retractability of the foreskin

The patients were evaluated every 15 days for a period of 6 weeks after treatment initiation by using the classification of Kikiros and Woodward by a single pediatric surgeon. The response to topical steroid treatment was defined as full retraction of the foreskin, i.e. Kikiros retractability grade 0 or 1 (Table I). Recurrence was defined as the reappearance of non-retractibility on stopping of the steroid. The presence of any possible local side effects of the steroid ointment was checked, including striae, pigmentation changes, telangiectasia, and hypertrichosis.

## Results

A total of 100 patients were retrospectively analyzed. The mean age at presentation was 3.9 years with the range being 1.5 years to 12 years. The most common complaints were inability to retract the prepuce, ballooning of the prepuce (77%) and diffculty in micturition (59%). Nineteen patients had grade 4 phimosis at first presentation. Resolution of symptoms and complete response was seen in 84 patients (84%). Sixteen patients did not show response to treatment. The patients who had a successful resolution of phimosis at 6 weeks were followed up after stopping the steroid for 6 weeks. Recurrence of the symptoms and non retractibility was seen in 3 patients. A total of 19 patients required surgical intervention.

## Discussion

It is necessary to differentiate between physiologic and pathologic phimosis in children to avoid unnecessary operative interventions. In children, physiologic phimosis is observed owing to adhesion between the inner foreskin and the glans penis [6]. At birth, approximately 96% of all males are known to have a non-retractile foreskin [1]. At the end of the first year, the retraction of the penile foreskin behind the glanular sulcus is possible in about 50% of boys; this increases to approximately 89% by the age of 3 years [7]. The incidence of phimosis decreases to about 8% by the age of 8 years to about 1% in males aged 16-18 years [7].

Physiologic phimosis consists of a pliant and unscarred preputial orifice. On the other hand, true pathologic phimosis exists when there is a failure to retract the penile foreskin which is secondary to distal scarring of the prepuce [8]. When applying gentle retraction to the normal but non-retractable infant foreskin, the distal part of the foreskin puckers and the narrow portion is proximal to the preputial tip. When the same gentle retraction is applied to the foreskin with true phimosis, it results in a cone-shaped foreskin with a fibrotic, circular band forming the most distal and narrowest part of the prepuce [9]. Pathological phimosis results when there are adherences to the fibrotic foreskin ring that make it impossible to expose the glans penis [10]. This alteration hinders adequate penile hygiene that favors foreskin infections, repeated urinary infections, sexually transmitted diseases and, at adult age, penile carcinoma [10]. The natural process of enlargement of the prepuce can suffer alterations when facing episodes of balanoposthitis. The traction of the preputial ring leads to the formation of a healing fibrosis and the impossibility to expose the glans [11].

Parents are often overtly anxious and concerned about non retractability in their children. Most of these cases end up in surgical interventions in form of circumcision. The operation of circumcision is not devoid of adverse effects and moreover has a huge economic impact. In order to avoid such surgeries, it is important to know about newer noninvasive, cheaper and safer treatment options.

Recently, i.e. in the past two decades, topical corticosteroids have been proposed as an alternative to surgery for the treatment of phimosis. The clinical treatment of phimosis with topical corticosteroids is a simple procedure, presents low costs and risks, is well accepted by the parents, has no major side effects and a good compliance to the treatment^3^. In this study, the parent compliance was good and there were no side effects.

Compared with placebo, corticosteroids significantly increase the complete or partial clinical resolution of phimosis [4]. Today circumcision is being reserved for recurrent balanoposthitis, pathological phimosis with a non-retractile, scarred foreskin, persistent physiological phimosis that is not resolved by other means and Balanitis Xerotica Obliterans [3].

Medium − to high-potency topical corticosteroids are effective for phimosis. A variable number of topical steroids have been proposed for topical use in phimosis like Triamcinolone, Hydrocortisone, Betamethasone, Clobetasone and Mometasone [3]. In this study, mometasone was used for topical application. The mechanism of the action of topical steroids in resolving phimosis remains speculative and multifactorial [5]. The first mechanism is related to an anti-inflammatory and immunosuppressive effect regulated by glucocorticoid activity; this stimulates the transcription of anti-inflammatory genes and decreases the transcription of inflammatory genes [6]. The humoral factors involved in the inflammatory response and leukocyte migration are inhibited. Glucocorticoids also interfere with the function of endothelial cells, granulocytes, and fibroblasts [6]. The second mechanism of topical steroids is related to a skin thinning effect caused by the inhibition of collagen synthesis [6]. Glucocorticoids inhibit the synthesis of hyaluronic acid, the main glycosaminoglycan produced by fibroblasts; thus the dermal extracellular matrix is reduced and collagen and elastin fibers become tightly packed and rearranged [6].

Varying results with success rates ranging from 67% to 95% have been reported [11]. Though side effects like suppression of the hypothalamo-pituitary-adrenal axis or cutaneous atrophy may occur; the doses used in the topical treatment of phimosis do not lead to this type of complications. The success rate in this study was 81% and there were no complications.

## Conclusion

The inability to retract the foreskin in a child may be physiologic or pathologic. For patients with physiological phimosis, local hygiene and parental reassurance is paramount. Topical steroid therapy accelerates the normal developmental process in these boys who would otherwise have been considered candidates for circumcision.
